# The impact of empathy on professional identity among Chinese junior male nurses: a moderated mediation model

**DOI:** 10.3389/fpsyg.2025.1389591

**Published:** 2025-02-12

**Authors:** Hezi Mu, Yi Cui, Lihua Zhang, Qin Liu, Lanfang Zhang, Haoshuang Yang, Changchang Chen, Na Liu, Yinling Zhang

**Affiliations:** ^1^Department of Nursing, Air Force Medical University, Xi’an, China; ^2^The Second Affiliated Hospital of Air Force Medical University, Xi’an, China

**Keywords:** junior male nurses, empathy, locomotion mode, emotional intelligence, professional identity

## Abstract

**Background:**

Nursing care is essential, but the role of junior male nurses in medical and health services is underestimated; thus, many junior male nurses leave the profession due to a lack of professional identity.

**Objective:**

This study examined how the mediating effect of emotional intelligence and the moderating role of locomotion mode influence the relationship between empathy and professional identity among Chinese junior male nurses.

**Methods:**

This cross-sectional descriptive study was conducted among junior male nurses in China from December 2021 to May 2022. We asked participants from ten hospitals to complete a questionnaire regarding empathy, emotional intelligence, locomotion mode, and professional identity. Bootstrap and simple slope methods were used to test the moderated mediation model.

**Results:**

Emotional intelligence partly mediated the effects of perspective-taking on professional identity (*β* = 0.253, *p* < 0.05). Furthermore, locomotion mode moderated the relationships between perspective-taking and emotional intelligence, perspective-taking and professional identity, and emotional intelligence and professional identity (*β* = 0.136, *p* < 0.01; *β* = 0.107, *p* < 0.05; *β* = −0.155, *p* < 0.01).

**Conclusion:**

The findings revealed that,the mediating effect of emotional intelligence on the relation between perspective-taking and professional adaptability was moderated by locomotion mode. Whereas. These findings are meaningful for early intervention and the improvement of professional identity among junior male nurses.

## 1 Background

China’s nursing shortage is becoming increasingly serious as a result of the country’s aging population, the major reform of the three-child policy, and increasing professional pressure. Relatively few men in China work in the nursing profession. By the end of 2021, the number of registered nurses in China had reached 5.019 million, but among those nurses, male nurses remained underrepresented, with the proportion of male nurses reaching only 3.8% (National Health Commission: China Health Statistics Yearbook, 2022). Although the proportion of male nurses has increased compared with the 2.3% recorded in 2019, the proportion is still relatively low (National Health Commission: China Health Statistics Yearbook, 2022). Junior male nurses might be inadequately represented in the study as a consequence of numerous social and cultural elements such as gender preconceptions, recruitment predicaments or more extensive systemic matters within the healthcare domain. Nursing has been considered an exclusively female profession (Chen et al., 2020). With the advancement of the nursing profession and the implementation of hospital policies, junior male nurses, as a vital component of the nursing group, have been exerting an ever more salient role in clinical divisions. Meanwhile, empirical evidence indicates that the professional identity of junior nurses with fewer than four years of work experience is more vulnerable to fluctuations (Chen et al., 2020; Enberg et al., 2007; MacIntosh, 2003). Consequently, it is indispensable to explore the professional identity status of junior male nurses.

Professional identity in nursing is an evolving process that includes the degree of recognition that an individual holds regarding his or her occupation and its professional values (Fitzgerald, 2020). Research has found that nurses’ values, beliefs, motivations, interpersonal relationships, and social communication skills are all related to their professional identity (McCarthy et al., 2020). Accordingly, nurses possessing an attenuated professional identity might confront a range of adverse effects, encompassing dissatisfaction with their clinical undertakings, viewing nursing as less than an optimal profession, and even escalating their propensity to depart from the profession. A qualitative study revealed that the professional identity level of nurses was significantly lower than that of employees in other professions and that the identity crisis of male nursing students was particularly apparent (Megginson, 2008). However, professional identity also has a positive effect on nursing outcomes. A strong professional identity can creates pride and satisfaction in nurses and plays an important role in professional cohesion (Chan et al., 2014). Nurses with a strong professional identity are often satisfied with what they receive from nursing and thus have a greater sense of subjective well-being, which is beneficial for nurses when working and collaborating with doctors, patients and family caregivers. This, in turn, allows them to gain recognition and trust (Ren et al., 2021). Although numerous studies have been conducted on nurses’ professional identity, most current research, both in China and other countries, has focused on female nurses or nursing students and less on junior male nurses (Li et al., 2022; Yu et al., 2022).

Empathy, a multidimensional construct defined as the ability to take on and understand another’s perspective, share another’s emotional experiences, and offer a feeling of warmth and acceptance. It encompasses both cognitive empathy and affective empathy. Cognitive empathy refers to an individual’s ability to discern another person’s condition and infer regarding their viewpoints, cogitations, and emotions from a psychological vantage point, whereas affective empathy entails an individual’s concern for another person’s feelings (Smith, 2006; Davis, 1983). Essentially, empathy serves as an integral component of person-centered care that fosters harmonious relationships, enhances patient compliance and improves the overall quality of care for the patient. Given that nursing is a profession laden with moral responsibilities, and the decisions made by nurses carry an obligation to provide comfort while upholding the personal dignity of individuals in need of professional care and treatment. Previous studies have indicated that nursing staff frequently encounter situations that impact their empathy, often related to informed consent, the unethical behavior of colleagues, and organizational policies that conflict with patient needs (Wang et al., 2022). These conflicts can result in psychological distress fatigue, which leads to diminished job satisfaction and quality of care and may ultimately contribute to a decrease in nurses’ professional identity. A few study founds that nurses’ professional identity is positively correlated with empathy (Wang et al., 2022; Hu et al., 2023). In addition, in line with a study by Sang that established that empathy could have an indirect effect on professional identity through psychological resilience, which was higher according to nursing students with higher empathy ability (Sang et al., 2022). Empathy helps nurses deepen their understanding of their profession, makes it more difficult for them to leave their jobs and increases their expectations to perform well in their career, thus ultimately leading to the improvement of their professional identity (Wang et al., 2022). However, as the majority of prior research has focused predominantly on female nurses and nursing students, insufficient attention has been given to male nurses. Consequently, it is essential to analyze the relationships and mechanisms between empathy and professional identity within this demographic.

The cognitive-affective processing system (CAPS) provides a theoretical framework for this study and suggests that there are two processes between original information and behavior (Mischel and Shoda, 1995; Mischel and Shoda, 1998; Mischel and Shoda, 2008). One is the encoding process, in which the original information is input into the cognitive-affective unit for encoding and interpretation. The second is the process of behavior generation, which produces different cognitive, emotional, and behavioral results through the interaction of cognitive-affective units (Mischel and Shoda, 1995; Mischel and Shoda, 1998; Mischel and Shoda, 2008). The CAPS model also encompasses a related concept known as the personality system. The personality system is not merely a collection of isolated factors; rather, it represents an intricate relational network that comprises cognitive and emotional mediating units. In general, the personality system tends toward stability; however, it may undergo changes when situational characteristics activate these cognitive and emotional units, ultimately triggering processes related to overt behavior. Therefore, in this study, male nurses are regarded as individuals that combine rationality and sensibility, and the formation mechanism of professional identity is observed through the establishment cognition–emotion paths. To create a greater sense of identity, individuals can best match their situations with themselves through self-regulation techniques such as emotional intelligence and the regulation model.

Individuals differ in their ability to express empathy in response to environmental changes or challenges. Emotional intelligence refers to the ability of an individual to perceive, understand, and manage his or her own emotions and those of others and guide his or her thinking and actions according to various emotional information (Salovey and Mayer, 1990). Emotional intelligence plays a valuable role in effectively processing information in changing environments. Thus, we suggest that empathy may stimulate professional identity through emotional intelligence on the basis of the cognitive–affective system theory of personality. Given that emotional intelligence has been proposed as a potential factor that increases empathy among male nurses (Hajibabaee et al., 2018), empirical findings have shown that empathy is positively associated with emotional intelligence (Zhu et al., 2016; Shi and Du, 2020; Abe et al., 2018). Moreover, emotional intelligence enhances the professional motivation of individuals, and thereby transforms their intrinsic emotions into actionable behaviors expressed through verbal communication, nonverbal cues, and other means. Taken together, patients’ emotional reactions are, ultimately, impacted. Research has shown that nurses who possess a higher level of emotional intelligence control their emotions more effectively and reduce their emotional consumption (Chen et al., 2022). When nurses can reduce adverse effects such as burnout and occupational stress, they can better feel pleasure and satisfaction in their work, which ultimately affects their professional identity (Chen et al., 2022; Huang et al., 2019; Karimi et al., 2014; Nightingale et al., 2018). Based on the above discussion, it is reasonable to predict that emotional intelligence may mediate the relation between empathy and professional identity.

The extant literature in the field of nursing reveals a relatively dearth of exploration concerning individual differences in prior studies on nurses’ professional identity; while clinical nursing practice evinces that the individual factors pertinent to nurses exert a salient influence on their professional identity. Kruglanski et al. (2000) proposed regulation model theory, which incorporates two independent dimensions of self-regulation: locomotion mode and assessment mode. Different regulatory modes have different effects on psychological adjustment. Locomotion mode is a psychological and behavioral strategy that individuals adopt in self-regulation, and it is used for the purpose of changing a situation via a straightforward approach (Mugon et al., 2018; Panno et al., 2015). Individuals who are in locomotion mode are more focused on making something happen, which reflects their sense of progress toward their goals; thus, they are more likely to have high subjective well-being and a high level of optimism (Higgins et al., 2003; Hanke et al., 2018). The assessment mode delineates a psychological framework wherein individuals conduct an exhaustive comparison and assessment of all alternative options and behavioral strategies throughout the self-regulation process, aiming to derive optimal behavioral decisions. Locomotion mode is another focus in the study of protective factors for professional identity (Huang et al., 2019; Hammarström et al., 2019). One study had shown that the locomotion mode facilitates psychological adjustment, alleviating psychological stress and occupational burnout (Bélanger et al., 2015). Another cross-sectional survey study showed that locomotion mode can also have a significant effect on nurses’ job satisfaction (Liu et al., 2019). In general, nurses characterized by high locomotion mode exhibit the lower levels of job burnout (Liu et al., 2019). Moreover, some variables, including burnout, job satisfaction and work engagement, can mediate the correlation between emotional intelligence and professional identity (Xing, 2022). Therefore, we speculate that nurses presenting a high locomotion mode might evince enhanced emotional intelligence in order to maintain positive professional identity. Consequently, this study further introduces the moderating variable of “locomotion mode.” Specifically, we predict that locomotion mode moderates the relationship between empathy and professional identity through the mediator of emotional intelligence.

**Figure 1:**
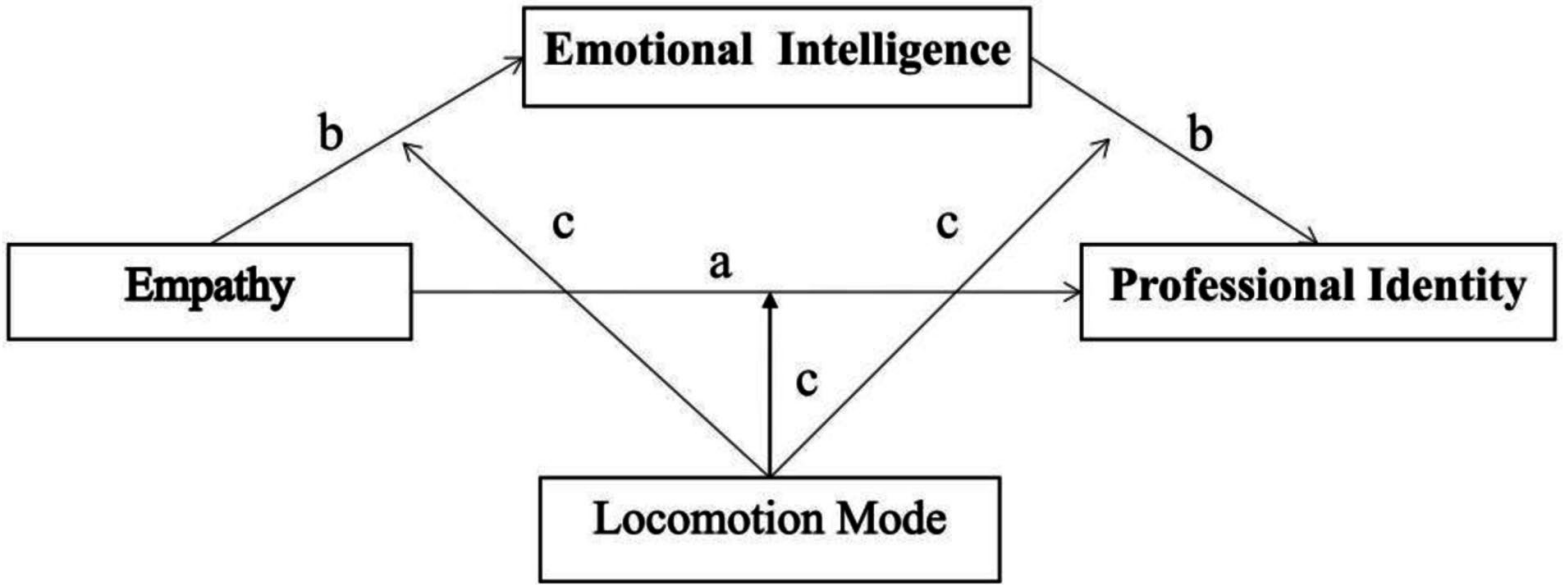
Schematic model of emotional intelligence as a mediator in the relationship between empathy and professional identity and locomotion mode as a moderator of the mediation.

In summary, drawing from classical theories, such as CAPS and the regulation model theory, the purpose of this study is to explore the influence of empathy on professional identity with a view to further improvement. Specifically, we aimed to investigate (a) whether empathy positively predicts professional identity and (b) whether emotional intelligence mediates the relationship between empathy and professional identity and (c) whether locomotion mode moderates the indirect relations between empathy and professional identity (see Figure 1).

## 2 Methods

### 2.1 Study design

This cross-sectional descriptive study was conducted among male nurses in China from December 2021 to May 2022. We used a convenience sampling method to select male nurses from 10 hospitals located in Henan, Shandong, Zhejiang, and Shaanxi provinces and asked them to complete a questionnaire regarding empathy, emotional intelligence, locomotion mode, and professional identity with the assistance of nurse managers.

### 2.2 Data collection and participants

According to Kendall’s principle of sample estimation, the sample size should be 5 ~ 10 times the number of questionnaire variables. There were 19 variables in total in this survey, thus the calculated sample size ranged from 95 to 190. Considering incomplete questionnaires and the loss of samples, we expanded the sample size by 20%; consequently, the required minimal sample size was 114.

In the present research, the inclusion criteria were as follows: junior male nurses (i) who had obtained the People’s Republic of China Nurse Practitioner Certification (there is a unified nurse registration system in China). A nurse who has been registered for practice and has obtained a nurse practitioner, certificate engage in nursing work in accordance with his or her registered place of practice [for more details, please refer to the relevant regulations (The Central People’s Government of the People’s Republic of China, 2021)]; (ii) who could independently engage in and take responsibility for clinical nursing work; and (iii) who were willing to participate in this study.

The exclusion criteria were as follows: junior male nurses (i) who were not in a nursing position during the investigation due to vacation, continuing education or other factors or (ii) who were in nonfront-line departments. Finally, of the 250 nurses to whom questionnaire packs were distributed, we recruited 226 junior male nurses who met the criteria. The return and valid response rates were both 90.4%.

### 2.3 Measurements

#### 2.3.1 Sociodemographic characteristics

We designed a sociodemographic characteristics questionnaire that consists of 6 questions on age, family location, family ambience, attitude toward nursing, job satisfaction, knowledge of empathy, and history of training in empathy.

#### 2.3.2 Regulatory mode scale

The Regulatory Mode Scale (RMS), developed by Kruglanski et al. (2000), was used to assess individuals’ self-regulation patterns. The Chinese version of the RMS (RMS-C) was translated and revised by Pang et al. (2012). The RMS-C contains 24 items in two dimensions: locomotion mode (12 items) and assessment mode (12 items). The items are measured on a 6-point Likert scale ranging from 1 (strongly disagree) to 6 (strongly agree). Items 2, 10, 12, 19, and 21 are appraised in an inverse scoring manner. A dimension score was computed by summing the twelve items separately, and the score ranged from 12 to 72. Higher scores indicated higher levels of locomotion or assessment mode. The two dimensions’ Cronbach’s *α* coefficients were 0.81 and 0.76, respectively.

#### 2.3.3 Nursing professional identity scale

The Nurses’ Professional Identity Scale (PIS), which was developed by Liu (Liu et al., 2011), was used to evaluate the level of professional identification among nurses. The questionnaire has 30 items that measure five components: professional cognitive assessment (9 items), professional social skills (6 items), professional social support (9 items), professional frustration response (6 items), and professional self-reflection (3 items). The items are measured on a 5-point Likert scale ranging from 1 (strongly disagree) to 5 (strongly agree). The total score ranges from 30 to 180, with higher scores indicating higher professional identity. The Cronbach’s *α* coefficient was 0.938.

#### 2.3.4 Interpersonal reactivity index scale

The Interpersonal Reactivity Index (IRI) was used to measure the state of nurses’ empathy. It was developed by Davis (1980), translated into Chinese and revised by Zhang et al. (2010). The IRI-C consists of 22 items divided into four subscales: perspective-taking (PT, 5 items), empathic concern (EC, 6 items), personal distress (PD, 5 items) and fantasy (FS, 6 items). The items are rated on a 5-point Likert scale ranging from 0 (does not describe me well) to 4 (describes me very well). Items 2, 5, 10, 11, and 14 are appraised in an inverse scoring manner. This study concentrates on emotional empathy and cognitive empathy; therefore, only the empathic concern and perspective-taking subscales of the IRI were selected for analysis. The Cronbach’s *α* coefficient of perspective-taking and empathic concern were 0.745 and 0.773, respectively.

#### 2.3.5 Wong and Law emotional intelligence scale

The Wong and Law Emotional Intelligence Scale (WLEIS) was used to measure the ability of nurses to evaluate and manage their personal emotions and respond to others’ emotions. It was developed by Wong and Law (2002) and translated and revised into Chinese by Wang (2010). There are 16 items on the scale across four dimensions: perception of one’s own emotions (12 items), managing one’s own emotions (8 items), appraising others’ emotions (6 items) and utilizing emotions (7 items). A 7-point Likert scale from 0 (strongly disagree) to 6 (strongly agree) is used for each item. The total score ranges from 0 to 96, with higher scores indicating higher emotional intelligence levels. The Cronbach’s α coefficient was 0.87.

#### 2.3.6 Data analysis

SPSS 26.0 and SPSS PROCESS macro software were used for data analysis and structural equation modeling (SEM). Descriptive analyses of sociodemographic characteristics were performed, including frequency, percentage, mean, and standard deviation (SD). The correlations among empathy (perspective-taking/empathic concern), regulatory mode, emotional intelligence, and nursing professional identity in junior male nurses were examined via Pearson correlations. Moreover, we used the Process Macro to examine the mediation and interaction effects. The significance level (two-tailed) was set at *p* < 0.05.

## 3 Results

### 3.1 Descriptive results

The sample and sociodemographic characteristics of the 226 junior male nurses are shown in Table 1. The participants’ ages ranged from 19 to 23 years, with a mean age of 21.10 ± 0.68 years. All the junior male nurses had 3 years of college education, and they had all graduated in the current year and has less than 1 year of clinical experience. The vast majority of the participants (87.17%) had a positive attitude toward nursing education. Approximately 76.1% of the junior male nurses liked nursing, and none disliked nursing. Finally, slightly more than half of the junior male nurses in this study reported that they had heard of empathy (51.77%) and had been trained (50.88%) in a clinic before working independently.

**Table 1:** Sociodemographic characteristics of junior male nurses [*N* = 226, cases and percentages (%)].

Variable	Category	*N*	%
Family location			
	City	65	28.76
	County & Town	54	23.89
	Rural	107	47.35
Family ambience			
	Harmony	193	85.40
	General	33	14.60
	Conflict	0	0
Attitude toward nursing			
	Favorite	172	76.11
	General	54	23.89
	Dislike	0	0
Job satisfaction			
	Very dissatisfied	8	3.5
	General	6	2.7
	Basically satisfied	58	25.7
	Very satisfied	154	68.1
Knowledge of empathy			
	None	109	48.23
	Little	76	33.63
	Substantial	41	18.14
History of training in empathy			
	No	111	49.12
	Yes	115	50.88

### 3.2 Correlation analysis

Table 2 shows the correlations among empathy, emotional intelligence, locomotion mode, and professional identity among junior male nurses. Professional identity was significantly positively correlated with perspective-taking, locomotion mode, and emotional intelligence (*r* = 0.536, *p* < 0.01; *r* = 0.553; *p* < 0.01; *r* = 0.662; *p* < 0.01), nonetheless, no substantive correlation was discerned with empathic concern (*r* = 0.071, *P* > 0.05). Additionally, a significant positive correlation was identified between perspective-taking and emotional intelligence (*r* = 0.482, *p* < 0.01), whereas no significant correlation was found with empathic concern (*r* = 0.111, *P* > 0.05). Locomotion mode was also significantly associated with perspective-taking, empathic concern, and emotional intelligence (*r* = 0.336, *p* < 0.01; *r* = 0.186, *p* < 0.01; *r* = 0.384, *p* < 0.01). See Table 2.

**Table 2:** Descriptive statistics and correlations among the variables (*n* = 226).

Variables	M ± SD	1. PIS	2.PCA	3.PSS 1	4.PSS 2	5.PFR	6.PSR	7.RMS	8. LM	9. AM	10. PT	11. EC	12. EI	13. POE	14. AOE	15. MOE	16. UE
1. PIS	123.03 ± 15.33	-	-	-	-	-	-	-	-	-	-	-	-	-	-	-	-
2.PCA	36.31 ± 5.48	0.906**	-	-	-	-	-	-	-	-	-	-	-	-	-	-	-
3.PSS 1	25.19 ± 3.10	0.848**	0.665**	-	-	-	-	-	-	-	-	-	-	-	-	-	-
4.PSS 2	23.19 ± 3.84	0.872**	0.700**	0.679**	-	-	-	-	-	-	-	-	-	-	-	-	-
5.PFR	25.65 ± 3.21	0.919**	0.782**	0.751**	0.763**	-	-	-	-	-	-	-	-	-	-	-	-
6.PSR	12.69 ± 1.81	0.800**	0.642**	0.686**	0.636**	0.739**	-	-	-	-	-	-	-	-	-	-	-
7.RMS	92.69 ± 9.91	0.266**	0.127	0.313**	0.338**	0.224**	0.222**	-	-	-	-	-	-	-	-	-	-
8. LM	52.46 ± 5.50	0.553**	0.413**	0.510**	0.566**	0.523**	0.432**	0.735**	-	-	-	-	-	-	-	-	-
9. AM	40.23 ± 6.95	−0.059	−0.147*	0.042	0.033	−0.095	−0.026	0.844**	0.256**	-	-	-	-	-	-	-	-
10. PT	13.81 ± 3.81	0.536**	0.446**	0.440**	0.480**	0.550**	0.445**	0.154*	0.336**	−0.047	-	-	-	-	-	-	-
11. EC	10.54 ± 2.55	0.071	0.000	0.178**	0.033	0.039	0.161*	0.216**	0.186**	0.161*	0.238**	-	-	-	-	-	-
12. EI	64.74 ± 9.35	0.662**	0.538**	0.545**	0.663**	0.619**	0.542**	0.223**	0.384**	0.013	0.482**	0.111	-	-	-	-	-
13. POE	16.96 ± 2.51	0.560**	0.472**	0.484**	0.538**	0.502**	0.458**	0.152*	0.286**	−0.009	0.454**	0.123	0.809**	-	-	-	-
14. AOE	15.90 ± 3.29	0.435**	0.275**	0.432**	0.440**	0.420**	0.440**	0.208**	0.308**	0.053	0.352**	0.229**	0.728**	0.471**	-	-	-
15. MOE	15.52 ± 3.04	0.519**	0.435**	0.366**	0.590**	0.470**	0.367**	0.077	0.234**	−0.076	0.402**	0.014	0.760**	0.595**	0.361**	-	-
16. UE	16.35 ± 3.44	0.509**	0.459**	0.397**	0.456**	0.495**	0.397**	0.237**	0.340**	0.069	0.283**	−0.004	0.756**	0.485**	0.407**	0.357**	-

*N = 226.*

PIS, professional identity scale; PCA, professional cognitive assessment; PSS-1, professional social skills; PSS-2, professional social support; PFR, professional frustration response; PSR, professional self-reflection; LM, locomotion mode; AM, assessment mode; EI, emotional intelligence; POE, perception of one’s own emotions; MOE, managing one’s own emotions; AOE, appraising others’ emotions; UE, utilizing emotions.

*Significant at *p* < 0.05; **Significant at *p* < 0.01.

### 3.3 Testing the hypothesized model and parameter estimates

On the basis of the test for conditional process analysis proposed by Hayes, we used the bootstrap method and carried out 5,000 bootstrappings for deviation correction to calculate the confidence intervals of the mediation and moderation analyses and standardized the data.

We used the SPSS macro PROCESS (Model 4) to conduct the mediation analysis. As shown in Table 3. In the bias-corrected percentile bootstrap analysis, we found that the direct effect and indirect effect were significant (*B* = 0.283, *p* < 0 0.05, 95% CI: 0.176, 0.389; *B* = 0. 253, *p* < 0.05, 95% CI: 0.160, 0.359), and the indirect effect accounted for 47.28% of the total effect. Therefore, emotional intelligence was found to partly mediate the relationship between perspective-taking and professional identity (see Table 3).

**Table 3:** Mediation test.

	Standardized effect	SE	95% CI LL	95% CI UL
Total effect	0.536	0.056	0.425	0.647
Direct effect	0.283	0.054	0.176	0.389
Indirect effect	0.253	0.051	0.160	0.359

**Figure 2:**
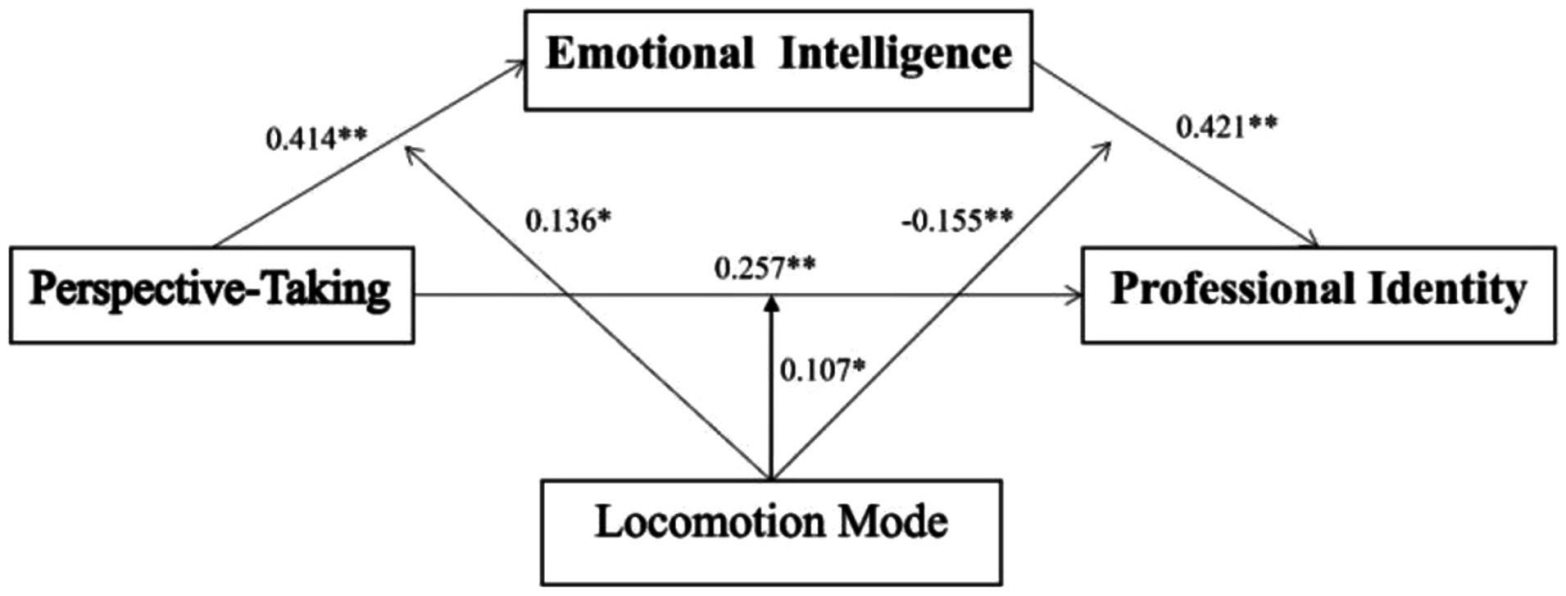
Final moderated mediation model.

We used the SPSS macro program PROCESS (Model 59) to conduct a moderated mediation analysis. The interaction effect of perspective-taking and locomotion mode for emotional intelligence was significant (*B* = 0.136, *p* < 0.05, 95% CI: 0.033, 0.239), Similarly, the interaction effect of perspective-taking and locomotion mode for professional identity was also significant (*B* = 0.107, *p* < 0.05, 95% CI: 0.011, 0.203), as was the interaction effect of emotional intelligence and locomotion mode for professional identity (*B* = −0.155, *p* < 0.05, 95% CI: −0.256, −0.053), which indicates that the relationships between perspective-taking and emotional intelligence, emotional intelligence and professional identity, and perspective-taking and professional identity are all moderated by locomotion mode. The final moderated mediation model is displayed in Table 4 and Figure 2. By analyzing the data, we found that empathic concern had no effect; therfore, we do not show this fingding here.

**Table 4:** Conditional process analysis.

	Emotional intelligence				Professional identity			
	*B*	*SE*	*t*	*P*	*B*	*SE*	*t*	*P*
Constant	−0.046	0.058	−0.782	0.435	0.023	0.046	0.502	0.616
Perspective-taking	0.414	0.060	6.956	<0.001	0.257	0.052	4.995	<0.001
LM	0.226	0.060	3.775	<0.001	0.322	0.048	6.743	<0.001
Perspective-taking× LM	0.136	0.052	2.610	0.010	0.107	0.049	2.204	0.029
EI	-	-	-	-	0.421	0.052	8.145	<0.001
EI× LM	-	-	-	-	−0.155	0.052	−2.987	0.003
*R^2^*	0.309				0.596			
*F*	33.107				64.947			

To clarify the moderating effect, the high and low groups were divided on the basis of 1 SD of locomotion mode, and the regression slopes of perspective-taking on emotional intelligence, perspective-taking on professional identity, and emotional intelligence on professional identity were tested in each group, i.e., simple slope test.

**Figure 3:**
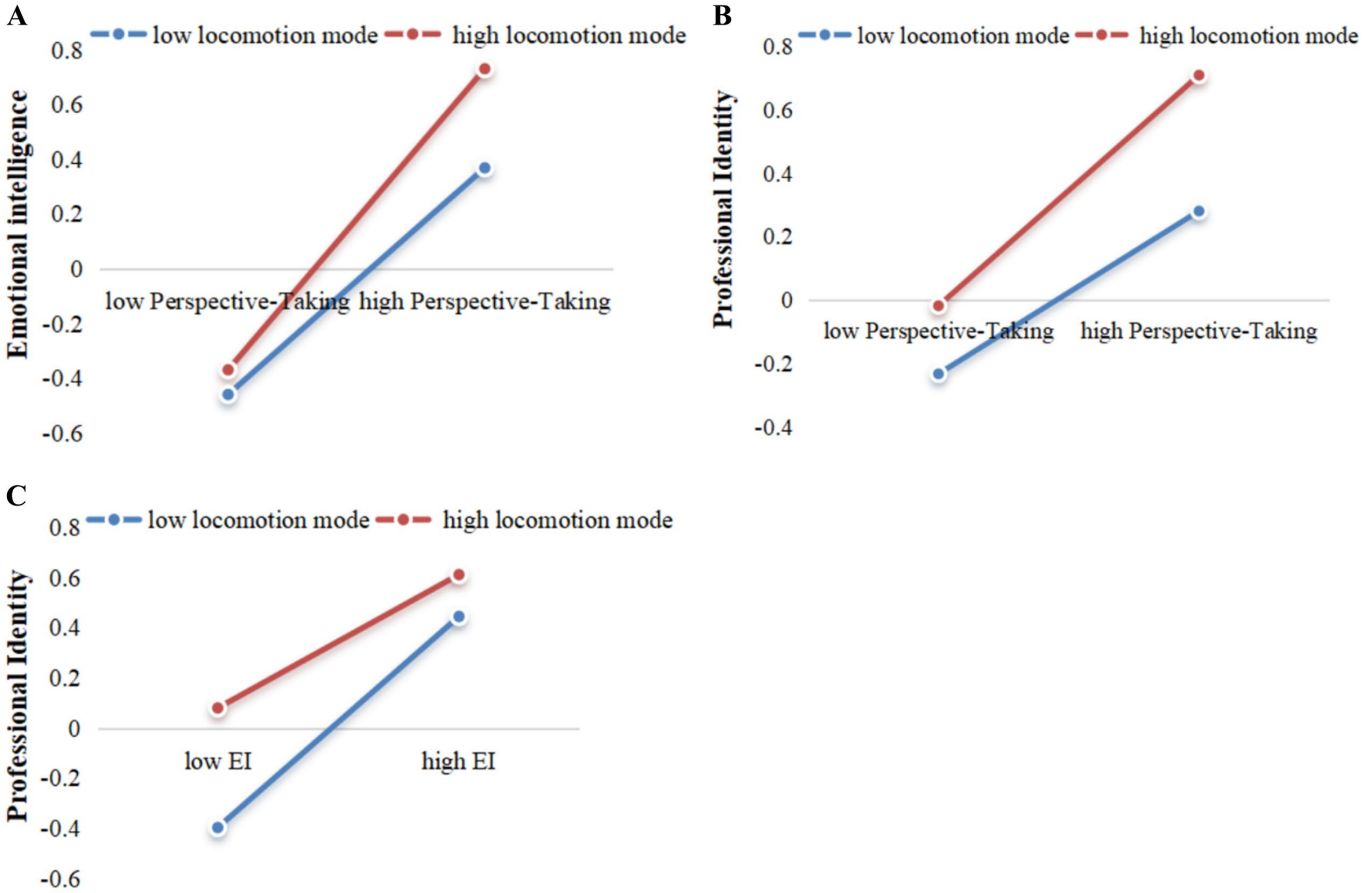
Moderating effects of locomotion mode on the relationships between perspective-taking and emotional intelligence, perspective-taking and professional identity, and emotional intelligence and professional identity.

The simple slope tests further suggested that when locomotion mode was low, perspective-taking was positively associated with emotional intelligence (*B* = 0.278, *p* < 0.001, 95% CI: 0.130, 0.426). At a high level, the association between perspective-taking and emotional intelligence remained significant (*B* = 0.551, *p* < 0.001, 95% CI: 0.387, 0.715). This result indicates that locomotion mode moderates the relationship between perspective-taking and emotional intelligence (see Figure 3A).

The simple slope tests suggested that when locomotion mode was low, perspective-taking was positively associated with professional identity (*B* = 0.150, *p* < 0.05, 95% CI: 0.029, 0.271), and when it was high, the association was still significant (*B* = 0.364, *p* < 0.001, 95% CI: 0.209, 0.520). This result indicates that locomotion mode moderates the relationship between perspective-taking and professional identity (see Figure 3B).

When locomotion mode was low, emotional intelligence was positively associated with professional identity (*B* = 0.575, *p* < 0.001, 95% CI: 0.435, 0.712), and with a high level of locomotion mode, the association between emotional intelligence and professional identity was still significant but weaker (*B* = 0.266, *p* < 0 0.001, 95% CI: 0.118, 0.414). This result indicates that locomotion mode moderates the relationship between emotional intelligence and professional identity (see Figure 3C).

In the path of Perspective-Taking→Emotional Intelligence→Professional Identity, locomotion mode had a moderating effect, indicating the presence of a mediating moderation effect (see Table 5). For junior male nurses in the low and high locomotion modes, the mediating moderation effect was significant but weaker (*B* = 0.160, *p* < 0 0.05, 95% CI: 0.058, 0.260; *B* = 0.147, *p* < 0 0.05, 95% CI: 0.042, 0.281), while for nurses in the medium locomotion mode, the mediating moderation effect was reached its highest level. That is, locomotion mode can regulate the influence of perspective-taking on professional identity through the mediation of emotional intelligence.

**Table 5:** Mediating moderation effect.

Locomotion mode	*B*	SE	95% CI LL	95% CI UL
M-1SD	0.160	0.050	0.058	0.260
M	0.174	0.038	0.106	0.255
M + 1SD	0.147	0.062	0.042	0.281

## 4 Discussion

Based on previous empirical and theoretical evidence, this study constructed a moderated mediation model of professional identity, with emotional intelligence as a mediating variable and locomotion mode as a moderating variable. We investigated two specific questions: first, whether empathy affects professional identity among male nurses and, second, how empathy among junior male nurses predicts professional identity. The results have theoretical and practical implications for furthering our understanding of the relationship between empathy and professional identity.

First, this study investigated the relationship between empathy and professional identity among junior male nurses, and the results showed that professional identity is positively correlated with perspective-taking, while no substantial correlation was identified with empathic concern, supporting our first hypothesis. Specifically, higher levels of perspective-taking (empathy) were linked to an increased likelihood of improving in professional identity. This result supports the conclusions of previous similar studies (Du et al., 2020; Ding et al., 2020). In the current study, nearly half of the junior male nurses indicated familiarity with empathy care and reported having participated in some form of empathy training (51.77%; 50.88%). Furthermore, a substantial majority (93.8%) expressed positive satisfaction with their profession. A reasonable explanation is that individuals who voluntarily choose the nursing profession are more likely to develop a long-term attachment to their career. When junior male nurses first enter the hospital for the clinical work, they gain a more intuitive and practical experience than in college; they begin to truly understand the essence of clinical nursing and gradually “think, act, and feel like a nurse” (Liu et al., 2022). Moreover, as junior male nurses enhance their perspective-taking abilities and provide compassionate care to patients, they cultivate a positive professional self-concept encompassing beliefs, values, attitudes, which in turn strengthens their professional identity (Wang et al., 2022). Notably, this study was conducted during the COVID-19 pandemic, a period during which paramedics were often referred to online as “the most beautiful retrogrades,” an expression that proved highly inspiring. Consequently, junior male nurses may have developed a more positive perception of their chosen profession and experienced an enhanced sense of professional identity in their daily work. This finding not only enriches the applicability of CAPS model in the medicine domain, but also suggests that developing empathy care and improving relationships among patients and nurses has implications for professional identify.

Second, we also reveals the mediating effect of emotional intelligence on the relationship between perspective-taking and professional adaptability, supporting our second hypothesis (Bas-Sarmiento et al., 2017; Štiglic et al., 2018). The formation of professional identity is a social process that develops in interactional relationships and in professional contexts (Wang et al., 2022). In the healthcare context, junior male nurses with high emotional intelligence are more competent in effectively identifying and comprehending both their own and patients’ emotions. Their communication with patients can achieve empathy and enhance communication efficiency, thereby strengthening the trust between nurses and patients and facilitating a harmonious nurse–patient relationship (Kılınç and Sis, 2021). Furthermore, they are capable of coordinating the relationship between life and work more effectively and are adept at leveraging emotional resources to discover more efficient methods for problem-solving. Additionally, the development of emotional intelligence can enhance the professional adaptability of individuals, mitigate burnout and work stress, and promote job satisfaction (Soto-Rubio et al., 2020; Soriano-Vázquez et al., 2023). When these emotions are recognized and understood, they are transformed into a more positive professional attitude, thereby reinforcing professional identity, which in turn guides their thinking and behavior later in life (Xing, 2022; He et al., 2022). Therefore, medical organizations should enhance junior male nurses’ emotional intelligence to improve their professional identity. A humanities course called “The Healer’s Art curriculum (HART)” and research on an empathy intervention called the “Knowledge, Simulation, and Sharing” (KSS) module similarly improved the professional identity of male nurses (Ding et al., 2020; Lawrence et al., 2020).

Finally, we also found that the mediating effect of emotional intelligence on the relation between perspective-taking and professional adaptability was moderated by locomotion mode. Specifically, the indirect effect of emotional intelligence was significant in junior male nurses with low, medium, and high locomotion mode, with the highest values observed in the medium locomotion mode. Locomotion mode is characterized by the pursuit of a change of state (Panno et al., 2015; Garcia et al., 2015). Existing research found that nurses who tried to choose locomotion in regulator mode had a stronger sense of job satisfaction, acquired sufficient self-confidence and optimism, and became more driven to work (Chernikova et al., 2016), which resulted in high professional identity. When adopting a medium level of locomotion mode, junior male nurses who neither exert excessive pressure on themself nor compromise their standards significantly experiences well-being and maintain positive expectations regarding their life and career. This approach facilitates a deeper understanding of the nursing profession, thereby enhancing their ability to perceive and manage affectivity as well as interpersonal relationships (Di Santo et al., 2023). Previous research has directly or indirectly found that locomotion mode is positively correlated with well-being, optimism, psychological vitality, and self-esteem (DeCarlo et al., 2014; Lucidi et al., 2016), which promotes the development of professional identity. Therefore, nurse managers should practice humanistic management, cultivate the career interests, educate them on career planning in a way that creates the space for career development, thus foster their professional identity.

## 5 Limitations

Several limitations should be considered when interpreting the findings of this study. First, we used a cross-sectional survey to analyze the professional identity of junior male nurses, thus limiting the possible to uncover a bidirectional relationship among empathy, emotional intelligence, and professional identity. Accordingly, further randomized controlled trials or longitudinal studies are needed in the future to validate this association. Second, our variables were all collected via self-administered questionnaires, and the results were inevitably affected by social desirability biases and may not be sufficiently objective. Consequently, future studies should collect multiple types of data, such as electroencephalograph recordings or third-party scorers. Quantitative data can also be seamlessly fused with qualitative data garnered via observations and interviews. Finally, our participants were recruited from four provinces and 10 hospitals, and thus, they are not nationally representative, and moreover, the participants included only clinically registered junior male nurses in China. Future research should employ larger-scale random sampling methodologies to investigate the professional identity of junior male nurses across diverse age groups while also integrating cultural and geographical factors.

## 6 Implications

Despite certain limitations, this study has several implications for theory and practice. For theoretical implications, this study extends the existing literature on empathy and professional identity in junior male nurses. Specifically, this study identified the mediating role of emotional intelligence in the relationship between perspective-taking and professional identity and illustrates when and how the mediation model becomes stronger or weaker and deepens and expands the mediation model, which is referred to as the moderating role of locomotion mode. Overall, the findings of this study add novel contributions to maintaining the empathy of nursing teams and increasing nurses’ professional identity.

With respect to practical contributions, nursing management aims to determine the level professional identity of male nurses in their daily clinical work. This may prompt them to have an initial understanding of how to engage in heart-to-heart talks and share their thoughts with others. The results of our study support the development and implementation of intervention programs for the professional identity of Chinese male nurses. First, nursing administrators should have an early understanding of the level of professional identity of male nurses, should pay more attention to junior male nurses’ mental health and psychosocial health, and should try to meet their external needs, as related to the work environment, wages, and personal development training. Second, nursing management is obligated to improve the working environment. A harmonious and supportive working atmosphere can enhances mutual understanding, improves subjective well-being, fosters greater empathy in the workplace, and empowers junior male nurses to effectively regulate their emotions. Finally, nurse managers should increase the work autonomy of nurses, develop training programs from multiple perspectives according to the characteristics of each nurse, and train them to become the nurses they strive to be, such as specialist nurses, clinical nursing instructors, nursing managers, etc. Regular mental health seminars should be implemented to promote engaging activities centered on emotional intelligence, thereby enabling individuals to accurately recognize their emotions and apply a regulation mode to effectively manage the diverse pressures inherent in clinical practice. This approach has the potential to invigorate enthusiasm for the nursing profession, bolster confidence in professional roles, and ultimately enhance professional identity.

## 7 Conclusion

This research examined the effects of emotional intelligence and locomotion mode on the relationship between empathy and professional identity among Chinese junior male nurses while consolidating previous professional identity studies. Emotional intelligence mediated the relationship between perspective-taking and professional identity, whereas locomotion mode moderated the three halves of the path in the mediating model of emotional intelligence. Nursing managers should pay attention to the status of the professional identity of junior male nurses and should adopt reasonable management strategies to increase the level of empathy, increase the capacity for emotional intelligence, apply locomotion mode to manage the diverse statuses, and improve the professional identity of junior male nurses. Further studies should continue to explore internal and external factors related to junior male nurses’ professional identities, formulate measures for maximizing these identities, and improve the reality of the individuals.

## Data Availability

The raw data supporting the conclusions of this article will be made available by the authors, without undue reservation.

## References

[ref1] Abe K. Niwa M. Fujisaki K. Suzuki Y. (2018). Associations between emotional intelligence, empathy and personality in Japanese medical students. BMC Med. Educ. 18:47. Published 2018 Mar 27. doi: 10.1186/s12909-018-1165-7, PMID: 29587725 PMC5870303

[ref2] Bas-Sarmiento P. Fernández-Gutiérrez M. Baena-Baños M. Romero-Sánchez J. M. (2017). Efficacy of empathy training in nursing students: a quasi-experimental study. Nurse Educ. Today 59, 59–65. doi: 10.1016/j.nedt.2017.08.012, PMID: 28945994

[ref3] Bélanger J. J. Pierro A. Kruglanski A. W. Vallerand R. J. de Carlo N. Falco A. (2015). On feeling good at work: the role of regulatory mode and passion in psychological adjustment[J]. J. Appl. Soc. Psychol. 45, 319–329. doi: 10.1111/jasp.12298

[ref4] Chan Z. C. Lo K. K. Tse K. C. Wong W. W. (2014). Self-image of male nursing students in Hong Kong: multi-qualitative approaches. Am. J. Mens Health 8, 26–34. doi: 10.1177/1557988313488929, PMID: 23686685

[ref5] Chen Y. Zhang Y. Jin R. (2020). Professional identity of male nursing students in 3-year colleges and junior male nurses in China. Am. J. Mens Health 14:1557988320936583. doi: 10.1177/1557988320936583, PMID: 32703068 PMC7383711

[ref6] Chen X. Y. Zi J. H. Liu Z. Liu Q. Zhang L. Gao P. H. (2022). Mediating effect of emotion regulation strategies on nurse’ vicarious traumatization and professional identity in public health emergencies. Chin. J. Convalescent. Med. 4, 430–434. doi: 10.13517/j.cnki.ccm.2022.04.026

[ref7] Chernikova M. Destro C. L. Mauro R. Kruglanski A. W. Higgins E. T. (2016). Different strokes for different folks: effects of regulatory mode complementarity and task complexity on performance. Personal. Individ. Differ. 89, 134–142. doi: 10.1016/j.paid.2015.10.011

[ref8] Davis M. H. (1980). A multidimensional approach to individual differences in empathy. JASAS Catalog Selected Doc. Psychol. 10.

[ref9] Davis M. H. (1983). Measuring individual differences in empathy: evidence for a multidimensional approach. J. Pers. Soc. Psychol. 44, 113–126. doi: 10.1037/0022-3514.44.1.113

[ref10] DeCarlo N. A. Falco A. Pierro A. Dugas M. Kruglanski A. W. Higgins E. T. (2014). Regulatory mode and well-being in organizations. J. Appl. Soc. Psychol. 44, 725–738. doi: 10.1111/jasp.12263

[ref11] Di Santo D. Lo Destro C. Baldner C. Talamo A. Cabras C. Pierro A. (2023). The mediating role of narcissism in the effects of regulatory mode on positivity. Curr. Psychol. 42, 6768–6777. doi: 10.1007/s12144-021-02014-w, PMID: 34220174 PMC8235914

[ref12] Ding X. Wang L. Sun J. Li D. Y. Zheng B. Y. He S. W.. (2020). Effectiveness of empathy clinical education for children's nursing students: a quasi-experimental study. Nurse Educ. Today 85:104260. doi: 10.1016/j.nedt.2019.104260, PMID: 31778862

[ref13] Du X. L. Gao Z. L. Yu X. X. Song J. (2020). A quantitative and qualitative study on the professional identity of male nursing students from a gender perspective. Health Vocational Educ. 18, 106–108.

[ref14] Enberg B. Stenlund H. Sundelin G. Ohman A. (2007). Work satisfaction, career preferences and unpaid household work among recently graduated health-care professionals--a gender perspective. Scand. J. Caring Sci. 21, 169–177. doi: 10.1111/j.1471-6712.2007.00453.x 17559435

[ref15] Fitzgerald A. (2020). Professional identity: a concept analysis. Nurs. Forum 55, 447–472. doi: 10.1111/nuf.12450, PMID: 32249453

[ref16] Garcia D. Jimmefors A. Mousavi F. Adrianson L. Rosenberg P. Archer T. (2015). Self-regulatory mode (locomotion and assessment), well-being (subjective and psychological), and exercise behavior (frequency and intensity) in relation to high school pupils' academic achievement. Peer J. 3:e847. Published 2015 Apr 2. doi: 10.7717/peerj.847, PMID: 25861553 PMC4389278

[ref17] Hajibabaee F. Farahani M. A. Ameri Z. Salehi T. Hosseini F. (2018). The relationship between empathy and emotional intelligence among Iranian nursing students. Int. J. Med. Educ. 9, 239–243. doi: 10.5116/ijme.5b83.e2a5, PMID: 30244237 PMC6387768

[ref18] Hammarström L. Häggström M. Devik S. A. Hellzen O. (2019). Controlling emotions-nurses' lived experiences caring for patients in forensic psychiatry. Int. J. Qual. Stud. Health Well-being 14:1682911. doi: 10.1080/17482631.2019.1682911, PMID: 31645227 PMC6818121

[ref19] Hanke S. Rohmann E. Förster J. (2018). Relationships between narcissistic grandiosity, narcissistic vulnerability, regulatory focus, regulatory mode, and life-satisfaction: data from two surveys. Data Brief 21, 861–865. Published 2018 Oct 18. doi: 10.1016/j.dib.2018.10.042, PMID: 30426037 PMC6223186

[ref20] He C. Y. Wu N. Lu Y. Zhang H. M. Zhou W. J. Chang S. Y. (2022). The mediating role of perceived stress on emotional intelligence and professional identity among nursing. Chin. Gen. Pract. Nurs. 2, 141–145. doi: 10.12104/j.issn.1674-4748.2022.02.001

[ref21] Higgins E. T. Kruglanski A. W. Pierro A. (2003). Regulatory mode: locomotion and assessment as distinct orientations. Adv. Exp. Soc. Psychol. 35, 293–344. doi: 10.1016/S0065-2601(03)01005-0

[ref22] Hu M. Zhang Z. Ou Y. Zhang H. Zheng X. Wu Y.. (2023). Importance of the Nurses' empathy level in operating rooms. Altern. Ther. Health Med. 29, 107–111, PMID: 37023311

[ref23] Huang H. Liu L. Yang S. Cui X. Zhang J. Wu H. (2019). Effects of job conditions, occupational stress, and emotional intelligence on chronic fatigue among Chinese nurses: a cross-sectional study. Psychol. Res. Behav. Manag. 12, 351–360. doi: 10.2147/PRBM.S207283, PMID: 31191056 PMC6526330

[ref24] Karimi L. Leggat S. G. Donohue L. Farrell G. Couper G. E. (2014). Emotional rescue: the role of emotional intelligence and emotional labour on well-being and job-stress among community nurses. J. Adv. Nurs. 70, 176–186. doi: 10.1111/jan.12185, PMID: 23763612

[ref25] Kılınç T. Sis Ç. A. (2021). Relationship between the social support and psychological resilience levels perceived by nurses during the COVID-19 pandemic: a study from Turkey. Perspect. Psychiatr. Care 57, 1000–1008. doi: 10.1111/ppc.12648, PMID: 33073874

[ref26] Kruglanski A. W. Thompson E. P. Higgins E. T. Atash M. N. Pierro A. Shah J. Y.. (2000). To "do the right thing" or to "just do it": locomotion and assessment as distinct self-regulatory imperatives. J. Pers. Soc. Psychol. 79, 793–815. doi: 10.1037/0022-3514.79.5.793, PMID: 11079242

[ref27] Lawrence E. C. Carvour M. L. Camarata C. Andarsio E. Rabow M. W. (2020). Requiring the Healer's art curriculum to promote professional identity formation among medical students. J. Med. Humanit. 41, 531–541. doi: 10.1007/s10912-020-09649-z, PMID: 32748226

[ref28] Li L. Gao X. Wang Y. Zeng C. Hou L. Xi Q. (2022). Professional identity and supporting willingness of nurses during the COVID-19 epidemic in China. Jpn. J. Nurs. Sci. 19:e12487. doi: 10.1111/jjns.12487, PMID: 35347842 PMC9115074

[ref29] Liu L. Hao Y. F. Liu X. H. (2011). Development of professional identity scale for nurses. Nurs. J. Chin. PLA 28, 18–20. doi: 10.3969/j.issn.1008-9993.2011.03.006

[ref30] Liu N. Wu D. Liu Q. Cheng H. Du X. L. Zhang Y. L. (2019). Mediating effect of emotional labor strategy on regulatory mode and nurses’ job satisfaction. J. Nurs. 26, 68–71. doi: 10.16460/j.issn1008-9969.2019.01.068

[ref31] Liu Y. Yao C. Zhao S. Han P. Jiang J. Duan X. (2022). Perspective and experience of male nursing students in 3-year vocational college during their clinical practicum: a qualitative study in Shanghai, China. Front. Public Health 10:905200. doi: 10.3389/fpubh.2022.905200 35719664 PMC9197777

[ref32] Lucidi F. Pica G. Mallia L. Castrucci E. Manganelli S. Bélanger J. J.. (2016). Running away from stress: how regulatory modes prospectively affect athletes' stress through passion. Scand. J. Med. Sci. Sports 26, 703–711. doi: 10.1111/sms.12496, PMID: 26059847

[ref33] MacIntosh J. (2003). Reworking professional nursing identity. West. J. Nurs. Res. 25, 725–741. doi: 10.1177/0193945903252419, PMID: 14528619

[ref34] McCarthy C. Boniol M. Daniels K. Cometto G. Diallo K. Lawani A. D.. (2020). State of the World’s nursing 2020: Investing in education, jobs, and leadership. Geneva: World Health Organization.

[ref35] Megginson L. A. (2008). RN-BSN education: 21st century barriers and incentives. J. Nurs. Manag. 071116232228007–071116232228008. doi: 10.1111/j.1365-2934.2007.00784.x, PMID: 18211335

[ref36] Mischel W. Shoda Y. (1995). A cognitive-affective system theory of personality: Reconceptualizing situations, dispositions, dynamics, and invariance in personality structure. Psychol. Rev. 102, 246–268. doi: 10.1037/0033-295X.102.2.246, PMID: 7740090

[ref37] Mischel W. Shoda Y. (1998). Reconciling processing dynamics and personality dispositions. Annu. Rev. Psychol. 49, 229–258. doi: 10.1146/annurev.psych.49.1.229, PMID: 9496625

[ref38] Mischel W. Shoda Y. (2008). “Toward a unified theory of personality: integrating dispositions and processing dynamics within the cognitive-affective processing system” in Handbook of personality. eds. John O. P. Robins R. W. Pervin L. A.. 3rd ed (New York, NY: Guilford Press).

[ref39] Mugon J. Struk A. Danckert J. (2018). A failure to launch: regulatory modes and boredom proneness. Front. Psychol. 9:1126. Published 2018 Jul 17. doi: 10.3389/fpsyg.2018.01126, PMID: 30065675 PMC6056760

[ref40] National Health Commission: China Health Statistics Yearbook. (2022). Available at: http://www.nhc.gov.cn/ (Accessed 30 July, 2023).

[ref41] Nightingale S. Spiby H. Sheen K. Slade P. (2018). The impact of emotional intelligence in health care professionals on caring behaviour towards patients in clinical and long-term care settings: findings from an integrative review. Int. J. Nurs. Stud. 80, 106–117. doi: 10.1016/j.ijnurstu.2018.01.006, PMID: 29407344

[ref42] Pang X. M. Wang L. Xiao F. Q. Qi B. (2012). The relationship between counterfactual thinking and regret: the regulatory mode as a moderator. J Psychol Sci. 5, 1137–1143. doi: 10.16719/j.cnki.1671-6981.2012.05.024

[ref43] Panno A. Lauriola M. Pierro A. (2015). Regulatory mode and risk-taking: the mediating role of anticipated regret. PLoS One 10:e0143147. Published 2015 Nov 18. doi: 10.1371/journal.pone.0143147, PMID: 26580960 PMC4651368

[ref44] Ren Z. Zhang X. Sun Y. Li X. He M. Shi H.. (2021). Relationships of professional identity and psychological reward satisfaction with subjective well-being among Chinese nurses. J. Nurs. Manag. 29, 1508–1516. doi: 10.1111/jonm.13276, PMID: 33501740

[ref45] Salovey P. Mayer J. D. (1990). Emotional intelligence. Imagin. Cogn. Pers. 9, 185–211. doi: 10.2190/DUGG-P24E-52WK-6CDG

[ref46] Sang N. Zhu Z. Z. Wu L. Shi P. L. Wang L. W. Kan H. Y.. (2022). The mediating effect of psychological resilience on empathy and professional identity of Chinese nursing students: a structural equation model analysis. J. Prof. Nurs. 43, 53–60. doi: 10.1016/j.profnurs.2022.09.002, PMID: 36496245

[ref47] Shi M. Du T. (2020). Associations of emotional intelligence and gratitude with empathy in medical students. BMC Med. Educ. 20:116. Published 2020 Apr 17. doi: 10.1186/s12909-020-02041-4, PMID: 32303212 PMC7164156

[ref48] Smith A. (2006). Cognitive empathy and emotional empathy in human behavior and evolution. Psychol. Rec. 56, 3–21. doi: 10.1007/bf03395534

[ref49] Soriano-Vázquez I. Cajachagua Castro M. Morales-García W. C. (2023). Emotional intelligence as a predictor of job satisfaction: the mediating role of conflict management in nurses. Front. Public Health 11:1249020. Published 2023 Nov 10. doi: 10.3389/fpubh.2023.1249020, PMID: 38026285 PMC10667434

[ref50] Soto-Rubio A. Giménez-Espert M. D. C. Prado-Gascó V. (2020). Effect of emotional intelligence and psychosocial risks on burnout, job satisfaction, and Nurses' health during the COVID-19 pandemic. Int. J. Environ. Res. Public Health 17:7998. doi: 10.3390/ijerph17217998 33143172 PMC7663663

[ref51] Štiglic G. Cilar L. Novak Ž. Vrbnjak D. Stenhouse R. Snowden A.. (2018). Emotional intelligence among nursing students: findings from a cross-sectional study. Nurse Educ. Today 66, 33–38. doi: 10.1016/j.nedt.2018.03.028, PMID: 29655019

[ref52] The Central People’ s Government of the People’ s Republic of China: Regulations of the National Health Commission-Management of Nurse Practitioner Registration (2021). Available at: http://www.gov.cn/zhengce/2020-12/27/content_5574490.htm

[ref53] Wang Y F. (2010). Reliability and validity of Chinese version of emotional intelligence scale. Available at: https://kns-cnki-net-443.libadmin.webvpn.net.cn/kcms2/article/abstract

[ref54] Wang Q. Cao X. Du T. (2022). First-year nursing students' initial contact with the clinical learning environment: impacts on their empathy levels and perceptions of professional identity. BMC Nurs. 21:234. Published 2022 Aug 23. doi: 10.1186/s12912-022-01016-8, PMID: 35999595 PMC9400203

[ref55] Wang L. Li H. Chen Q. Fang C. Cao L. Zhu L. (2022). Mediating effect of workplace violence on the relationship between empathy and professional identity among nursing students. Front. Psychol. 13:964952. Published 2022 Dec 12. doi: 10.3389/fpsyg.2022.964952, PMID: 36578675 PMC9791219

[ref56] Wong C. S. Law K. S. (2002). The effects of leader and follower emotional intelligence on performance and attitude: an exploratory study. Leadersh. Q. 13, 243–274. doi: 10.1016/S1048-9843(02)00099-1

[ref57] Xing Z. (2022). English as a foreign language Teachers' work engagement, burnout, and their professional identity. Front. Psychol. 13:916079. Published 2022 Jun 9. doi: 10.3389/fpsyg.2022.916079, PMID: 35756227 PMC9218421

[ref58] Yu J. F. Ding Y. M. Jia R. Y. Liang D. D. Wu Z. Lu G. L.. (2022). Professional identity and emotional labour affect the relationship between perceived organisational justice and job performance among Chinese hospital nurses. J. Nurs. Manag. 30, 1252–1262. doi: 10.1111/jonm.13608, PMID: 35355353

[ref59] Zhang F. F. Dong Y. Wang K. Zhan Z. Y. Xie L. F. (2010). Reliability and validity of the Chinese version of the interpersonal reactivity index-C. Chin. J. Clin. Psychol. 18, 155–157. doi: 10.16128/j.cnki.1005-3611.2010.02.019

[ref60] Zhu C. Q. Lu Y. Dong B. L. (2016). The influence of emotional intelligence and emotion regulation on college students’ exercise involvement: a model of mediating effec. J Shandong Sport Univ. 2, 90–96. doi: 10.14104/j.cnki.1006-2076.2016.02.016

